# Field Evaluation of Low-Cost Particulate Matter Sensors for Measuring Wildfire Smoke

**DOI:** 10.3390/s20174796

**Published:** 2020-08-25

**Authors:** Amara L. Holder, Anna K. Mebust, Lauren A. Maghran, Michael R. McGown, Kathleen E. Stewart, Dena M. Vallano, Robert A. Elleman, Kirk R. Baker

**Affiliations:** 1 US Environmental Protection Agency, Office of Research and Development, Research Triangle Park, NC 27711, USA; 2 US Environmental Protection Agency, Region 9, San Francisco, CA 94105, USA; mebust.anna@epa.gov (A.K.M.); maghranlauren@gmail.com (L.A.M.); stewart.kathleen@epa.gov (K.E.S.); vallano.dena@epa.gov (D.M.V.); 3 US Environmental Protection Agency, Region 10, Seattle, CA 98101, USA; mcgown.michael@epa.gov (M.R.M.); elleman.robert@epa.gov (R.A.E.); 4 US Environmental Protection Agency, Office of Air Quality Planning and Standards, Research Triangle Park, NC 27711, USA; baker.kirk@epa.gov

**Keywords:** air quality, smoke, environmental monitoring

## Abstract

Until recently, air quality impacts from wildfires were predominantly determined based on data from permanent stationary regulatory air pollution monitors. However, low-cost particulate matter (PM) sensors are now widely used by the public as a source of air quality information during wildfires, although their performance during smoke impacted conditions has not been thoroughly evaluated. We collocated three types of low-cost fine PM (PM_2.5_) sensors with reference instruments near multiple fires in the western and eastern United States (maximum hourly PM_2.5_ = 295 µg/m^3^). Sensors were moderately to strongly correlated with reference instruments (hourly averaged r^2^ = 0.52–0.95), but overpredicted PM_2.5_ concentrations (normalized root mean square errors, NRMSE = 80–167%). We developed a correction equation for wildfire smoke that reduced the NRMSE to less than 27%. Correction equations were specific to each sensor package, demonstrating the impact of the physical configuration and the algorithm used to translate the size and count information into PM_2.5_ concentrations. These results suggest the low-cost sensors can fill in the large spatial gaps in monitoring networks near wildfires with mean absolute errors of less than 10 µg/m^3^ in the hourly PM_2.5_ concentrations when using a sensor-specific smoke correction equation.

## 1. Introduction

Fine particulate matter (PM smaller than 2.5 µm, PM_2.5_) is a major component of wildfire smoke. Exposure to wildfire PM_2.5_ has established adverse health effects and can be of special concern to sensitive populations [1]. Actions to mitigate the impact of wildfire smoke on public health include reducing outdoor physical activity, using portable air cleaners in homes and other indoor spaces, and opening clean air centers [2]. The use of these exposure mitigation strategies as well as other actions aimed at reducing smoke exposure—such as canceling outdoor events or closing schools and places of business—are contingent on knowing local pollutant concentrations to calculate the Air Quality Index (AQI). The AQI is typically based on pollutant concentrations measured by a stationary regulatory monitoring network. However, rural areas with more frequent wildfires also tend to have few to no regulatory monitors. Additionally, during smoke events, the nearest monitoring site may not accurately represent local pollution concentrations due to complex topography, localized smoke plumes, and drainage patterns that result in large spatial gradients in smoke concentrations [3]. During wildfire smoke episodes, state or local air quality agencies and the United States Forest Service (USFS) will often augment the stationary monitoring network with temporary monitoring sites [4]. However, large gaps remain in the spatial coverage of monitors in smoke-impacted areas, affecting the accuracy of the AQI in some areas.

The gaps in spatial coverage and increased options for low-cost air quality measurements has led to the proliferation of low-cost PM sensors—such as the PurpleAir and others—to provide localized information on air quality conditions during wildfires [5]. The increasing popularity of these sensors is demonstrated by widespread use in many parts of the U.S. and the news media using the PurpleAir map as a source for local estimates of PM_2.5_ concentrations during wildfires [6]. However, there is limited information on the performance of PM sensors during wildfires compared to the monitors currently used in temporary networks or at regulatory monitoring sites [7,8]. Therefore, the ability of a PM sensor to accurately represent smoke air quality impacts is unknown, limiting the utility of these low-cost sensors for public health decisions.

Over the past five years, there have been numerous evaluations of PM sensor performance that have covered a range of sensor manufacturers and models, PM sources, and environmental conditions. Most of these sensors are based on light scattering principles and are either optical particle counters that count and determine the size of particles using scattered light or nephelometers that translate scattered light by an ensemble of particles into PM mass [9,10]. Laboratory studies have shown that many PM sensors have a high correlation with a reference instrument, which is often an optical-based research-grade instrument calibrated to filter mass or a designated regulatory instrument that has been validated against filter measurements [10]. Additionally, some PM sensors maintain linearity at concentrations as high as 500 µg/m^3^ [7,11,12,13,14] with a quadratic response to concentrations as high as 10,000 µg/m^3^ [11,14]. However, sensor performance has varied by manufacturer and model, even when using the same original equipment manufacturer (OEM) sensor, demonstrating the importance of the physical configuration of the sensor package, sensor design (e.g., laser, light detector, flow path, etc.), and data processing algorithms [11,15,16,17,18]. 

Many sensors have been shown to reliably reproduce PM_2.5_ concentration trends, but report concentrations as much as 200% different from a reference measurement. Evaluations with different types of aerosols (incense, Arizona road dust, NaCl, etc.) have shown that sensor accuracy varies and particle size, chemical composition, and optical properties are the dominant factors determining the response [12,13,17,18,19]. PM sensors may have some difficulty measuring larger particles (e.g., PM larger than 2.5 µm), with poorer correlations (r^2^ = 0.1–0.3) observed during times when the crustal fraction of PM was elevated [8]. Additionally, environmental conditions can impact accuracy [20,21]. Some sensors have exhibited a high bias (up to 50%) with increasing temperature (T) [16] and increasing relative humidity (RH) (up to 90% when RH exceeded 75%) [7,10,20,21]. The sensitivity to RH may depend on particle composition, with PM hygroscopicity playing an important role [19,20]. The combined impact of changing environmental conditions and variable aerosol characteristics suggests that seasonal or source specific calibrations may improve the accuracy of low-cost sensors [8,22]. Field calibration, under conditions similar to the measurement application, is essential to obtain accurate measurements from low-cost PM sensors [9,10]. 

Although low-cost PM sensors have been widely used to assess the air quality impacts of wildfire smoke, there is minimal information on their performance under heavy smoke. During a long-term study in Salt Lake City, Sayahi et al. [8] observed a good correlation between a Plantower PMS5003 sensor and the reference instrument but reported PM_2.5_ concentrations over 1.5 times the reference concentration during wildfire smoke impacted times, unlike other times of the year when the sensor response was 0.3–1.25 times the reference. However, hourly smoke concentrations only reached ~60 µg/m^3^, limiting the assessment of sensor linearity at high concentrations. More recently, Delp and Singer [23] found that five different models of low-cost PM sensor provided accurate measurements with a linear response to wildfire smoke concentrations as high as 150 µg/m^3^, but they each required a correction factor that differed between sensors even when using the same OEM sensing unit (Plantower PMS5003). A laboratory study by Mehadi et al. [7] was able to achieve much higher concentrations (883 µg/m^3^) with simulated smoke in the evaluation of two low-cost sensors (PurpleAir and Dylos). They found a low correlation between the PurpleAir and the reference instrument (r^2^ = 0.33), and that the ratio of the sensor to reference PM_2.5_ concentrations varied from about 1.5 to 3.5 with increasing smoke concentration. This sensor-to-reference ratio also decreased as the smoke elemental carbon/organic carbon ratio increased, showing that sensor performance is sensitive to smoke composition. 

Therefore, there is still a need for field evaluation of low-cost PM sensors near wildfires to validate their performance under smoke impacted conditions. The objective of this study was to deploy several low-cost PM sensors in wildfire smoke impacted areas alongside reference instruments and evaluate their accuracy and linearity at high smoke concentrations.

## 2. Materials and Methods

### 2.1. Instruments

Low-cost PM sensors (<$5000 per unit) were selected based on either their prevalent use by the public or their applicability for temporary smoke monitoring networks. Only systems in weather-resistant enclosures were selected to ensure their suitability for extended outdoor monitoring. The sensors selected for this study were: (1) The SenSevere Real-Time Affordable Multi-Pollutant monitor (RAMP), (2) The Aeroqual micro air quality station (AQY), and (3) The Purple Air PA-II-SD (PA). Table S1 in the Supplementary Materials lists the sensor systems, the measured pollutants, and additional meteorological parameters (Temperature, RH, and wind speed and direction) measured by each system. After this study was carried out, the SenSevere Real-Time Affordable Multi-Pollutant monitor (RAMP) was declared the winner of a U.S. interagency challenge put forth by the U.S. Environmental Protection Agency (EPA) and others for the development of a low-cost option for measuring wildfire smoke [24]. The RAMP was evaluated with laboratory wildfire simulations for the challenge and had not been evaluated in the field. The AQY was selected as it is marketed to air quality agencies as a low-cost monitoring solution. The PA was included in the evaluation due to its widespread use among the public. 

PM_2.5_ was the only pollutant evaluated in this study because PM_2.5_ exposure is the greatest air quality hazard posed by wildfires. Furthermore, PM_2.5_ is frequently the only pollutant measured in the temporary air monitoring networks set up to monitor air quality impacts from wildfires and was the only reference measurement available at all locations. The PA, unlike the other sensors evaluated here, reports multiple PM_2.5_ measurements corresponding to the two identical sensors (a and b) and a correction factor (CF) for indoor (CF = 1) and outdoor (CF = atm) applications. For some versions of the PA firmware, there was an inconsistency between the PA CF = 1/atm label and the label reported by the OEM sensor used in the PA package (Plantower, Beijing, China, PMS5003) [25]. In this work, we refer to the data reported with the lower concentration here as CF = atm consistent with the PMS5003, despite being labeled as CF = 1 in the PA firmware (v2.50i) of the sensors used in this study. The PA data with the lower concentration is currently displayed on the public PA map. The ratio of the CF = atm PM_2.5_ channel to the CF = 1 channel shows a piecewise linear relationship between the two channels: a ratio of 1.0 below 25 μg/m^3^, a linear reduction in the ratio up to CF = 1 of 90 μg/m^3^, and a constant ratio of 0.66 at higher CF = 1 concentrations (Figure S2). 

To enable long-term field deployment, each sensor system was built into a portable, shipping/mounting container. Each system included a pole mount, solar panel, battery, and line power plug so the system could be deployed in areas with and without power access (Figure S1). The RAMP and the PA had onboard data storage, but the version of the AQY evaluated here required a network connection for data to be saved to the Aeroqual cloud service. Because the AQY was operated in areas without cellular service, the AQY was not connected to the Aeroqual Cloud, but rather to a computer (Raspberry Pi 3) for data logging. 

Sites were selected based on their likeliness of being impacted by wildfire smoke and the availability of a collocated PM_2.5_ measurement (Table 1). The sensors were also operated outside at the Ambient Innovation Research Site (AIRS) on the EPA Research Triangle Park (RTP) campus. Sensors were mounted on a deck approximately 2 m above ground either on the railing or on a pole. The collocated reference instrument was installed in an environmentally controlled shelter sampling through the roof located approximately 11 m from the sensors and approximately 3 m above ground. The sensors were collocated at the AIRS site in varying numbers and durations due to instrument failures, returns to the manufacturer, and threats from severe weather (e.g., hurricanes).

The collocated reference instrument (EDM180, GRIMM) at the AIRS site was operated by the EPA and is designated as a federal equivalent method (FEM) by the EPA for regulatory PM_2.5_ measurements. FEM reference measurements were desired, but not required, because FEM designations are validated against filter-based EPA federal reference methods and have been shown to provide accurate PM_2.5_ concentration measurements [26]. Collocated reference instruments at each fire were operated by the USFS, U. S. National Park Service, or the California Air Resources Board and consisted of both FEM instruments (BAM 1020, Met One) and those without an FEM designation (E-BAM, Met One and E-SAMPLER, Met One) that do not meet the regulatory requirements. These non-FEM instruments (E-BAM and E-SAMPLER) have been evaluated for their performance for wildfire smoke [27] and are often used for smoke monitoring for health messaging purposes.

### 2.2. Wildfire Deployments

Fire deployments were carried out in conjunction with the USFS Wildfire Air Quality Response Program (WFAQRP). WFAQRP Air Resource Advisors assigned to the wildfire deployed USFS instruments and the low-cost sensors in areas surrounding the wildfire. Generally, the sites were located at USFS buildings or fire stations within a 100-mile radius of the fire. These locations may have been briefly impacted by local sources, such as trucks or road dust, but any short duration PM sources would typically be overwhelmed by wildfire smoke, which was often present at elevated concentrations for several days or weeks. Sensors were sited within 3 m of the collocated reference instrument and mounted on a pole 1 to 1.5 m above ground. The sites were free of any potential obstructions to air flow and not located near trees. Most sites were near a roadway (~20 m away) and some roads were unpaved. Roads were infrequently traveled and no short duration spikes in concentration that may reflect road dust were observed in the sensor datasets.

### 2.3. Data Analysis

Collocated sensors were time-aligned and averaged on an hourly basis to match the typical time resolution of temporary smoke monitors. The hourly averaged datasets were compared using linear least-squares regression and a coefficient of determination describes the goodness of fit. The mean bias error (MBE), mean average error (MAE), root mean square error (RMSE), and normalized root mean square error (NRMSE) were calculated for each hourly dataset as described in the Appendix A. At the RTP location, 2–3 sensors were collocated from two to eight months (Table 2, Table 3 and Table 4), but the longer collocations were with only two sensors of each type. The average percent difference (PD_avg_) for two sensors of each type was used to evaluate the sensor precision as described in the Appendix A.

Linear multivariate regression was done to determine the impact of environmental parameters on the comparison between the various PM_2.5_ measurements and to create correction factors. The statistical significance of the linear correlation of sensor data with other sensors or the reference was determined with a t-test with *p*-value of 0.01 if not otherwise noted. A comparison of slopes and intercepts between linear regressions was done using an analysis of covariance (ANCOVA) with a *t*-test for significance for the coefficients [28].

## 3. Results and Discussion

**Figure 1:**
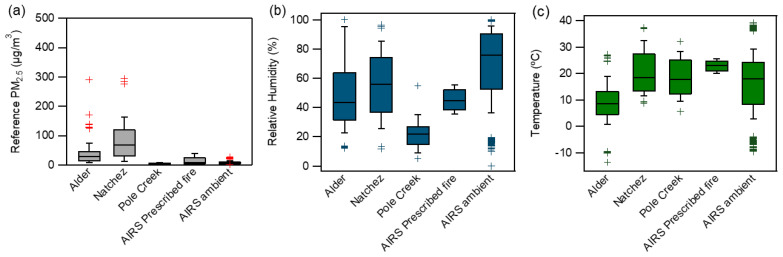
Box plots for (**a**) reference PM_2.5_ hourly concentrations (**b**) RH and (**c**) temperature at each fire from the reference monitor. Whiskers denote the 10th and 90th percentiles, symbols represent data outside of 1.5 times the interquartile range. Natchez RH and temperature estimated from corrected PA and AQY measurements.

The deployments captured a range of particle concentrations (Figure 1 and Table S2) spanning all PM_2.5_ AQI categories, ensuring a broad comparison of each PM sensor in conditions typical of areas impacted by wildfires. The dynamic nature of wildfire smoke plumes made siting difficult and not all deployment locations were impacted by high concentrations. For example, during the Pole Creek-Bald Mountain fire, the sensors and reference instruments were deployed at two different locations in the same region as the fires, but neither site was impacted by smoke with hourly PM_2.5_ concentrations remaining below 9 µg/m^3^. The highest recorded hourly PM_2.5_ concentration was 295 µg/m^3^ near the Natchez Fire.

### 3.1. Evaluation of Meteorological Measurements

All sensors measured temperature and RH but comparison measurements for most of these parameters were not available at all fires and the range of temperature and RH were limited (Figure 1b,c), so the primary evaluation of the meteorological measurements was done at the AIRS site where a range of ambient conditions were encountered. The AQY hourly temperature and RH measurements exhibited some of the closest agreements with the reference measurement of all the sensors tested here (Temperature r^2^ = 0.98 *p* < 0.001, MBE = 1.83 °C and RH r^2^ = 0.95 *p* < 0.001, MBE = −4.9% Table 2 and Table 3). Additionally, the two AQY sensors operated at AIRS had high precision with PD_avg_ of 1.1% and 11.4% for temperature and RH, respectively.

The PA hourly temperature and RH measurements, like the AQY, were strongly correlated with the reference values (average r^2^ = 0.91 and 0.84 respectively, *p* < 0.001) and had high precision (temperature PD_avg_ = 6.1%, RH PD_avg_ = 4.0%) (Table 2 and Table 3), but the absolute values were substantially different from the reference. The PA reported warmer and dryer conditions than the ambient reference (temperature MBE = 5.3 °C and RH MBE = −24.3%), which has been observed in other PA evaluations [21,22]. This deviation from the reference measurement is likely due to the location of the temperature and RH sensor inside the PA case where it is heated from the sensor electronics. Therefore, the PA temperature and RH measurement are interpreted as an internal rather than ambient measurement.

The RAMP hourly temperature had a high correlation (average r^2^ = 0.95, *p* < 0.001) with the reference measurement. Unlike the other sensors, the RAMP exhibited a diurnal trend with the temperature nearly 35% higher during daylight hours. The largest bias was +10 °C and was typically observed from 12:00 pm to about 3:00 pm. Overnight bias was 0 to −2 °C. We compared the bias with weather observations recorded at the Raleigh Durham Airport for several days in February, March, April, and July and found that lower daytime bias was associated with rainy weather, but the bias was still elevated under cloudy conditions. It is therefore possible that the higher measured temperature is partly due to radiant heating from the sun. The RAMP RH measurement was also highly correlated (r^2^ = 0.95, *p* < 0.001) with the reference but showed clear temporal deviations, often reporting 14% lower RH overnight but nearly the same RH during the daytime hours. Therefore, the RAMP temperature and RH do not reflect ambient values and may be a more suitable measure of internal conditions, which may require additional calibration. The RAMP temperature PD_avg_ was 4.8% and the RH PD_avg_ was 2.2% (Table 2 and Table 3). 

### 3.2. Evaluation of PM_2.5_ Measurement—Ambient

The hourly PM_2.5_ averages for the different sensors exhibited moderate to good correlations with the reference at the AIRS site (Table 4), despite very low PM_2.5_ concentrations (7.7 ± 4.3 µg/m^3^, mean and standard deviation). The AQY and the RAMP reported concentrations that were correlated with the reference, but with slopes of 0.89 and 0.88 that were significantly different (*p* < 0.001) from the reference (i.e. slope = 1), intercepts of −0.21 and 2.45 and MBEs of −0.01 and 1.64 µg/m^3^ respectively. The PA had a strong correlation with the reference but overreported PM_2.5_ concentrations with a statistically significant slope of 1.61 (*p* < 0.001), an intercept of −1.40, and MBE of 2.89 µg/m^3^ for CF=atm (slope of 1.63, an intercept of −1.51 and MBE of 2.92 µg/m^3^ for CF = 1, Table 4). The PA CF = atm and CF = 1 regressions for the AIRS site were not statistically different for the slope or the intercept since CF only differs when PA reads greater than 25 µg/m^3^, which happened infrequently at the AIRS site.

The PA and RAMP PM_2.5_ measurements exhibited low PD_avg_ throughout the AIRS collocation period (Table 4) and, when compared over the same sampling time period (12/2018–05/2019), had almost identical PD_avg_ (PA = 7.78%, RAMP = 7.94%), which is not surprising considering they use the same OEM sensor (PMS5003). The AQY, which has a different OEM sensor (Novafitness, Jinan, China, Model SDS011), had the highest variation across sensors due to one sensor reporting approximately half the concentration of the others. The AQY manual suggests that setting user-specified calibration factors (slope and offset) for the PM_2.5_ measurement will result in optimal data quality. The AQY were operated for this study using the calibration factor set by the manufacturer. The AQY reporting lower concentrations came with different manufacturer-set calibration factors than the other two sensors. When this AQY was left out of the comparison, the PD_avg_ was only 13.4% (Table 4), showing reasonable agreement between the two remaining AQY units. The AQY also exhibited a modest, but statistically significant (*p* < 0.001) correlation with the reference, with r^2^ of 0.39, 0.45, and 0.59 for the three units. At the low concentrations observed at the AIRS site, the AQYs exhibited a low bias and did not follow the diurnal PM_2.5_ concentration trends. 

### 3.3. Evaluation of PM_2.5_ Measurement–Smoke Impacted

**Figure 2:**
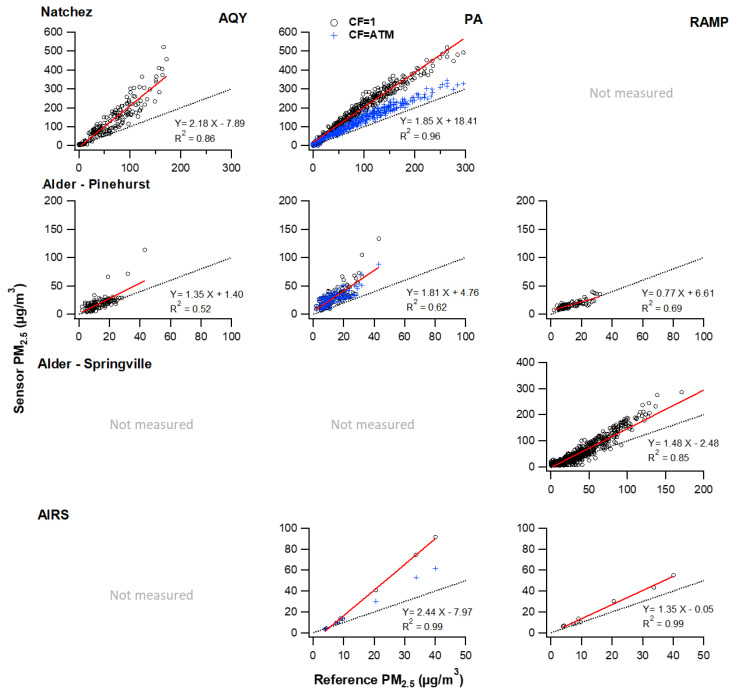
Scatter plot of sensor hourly averages compared to the reference for smoke impacted times. Red lines show linear least-squares fit; black lines show a one-to-one relationship. Columns correspond to the sensor type and rows correspond to the measurement location. Note the varying concentration range measured at each location.

Overall, the PM_2.5_ sensors evaluated here had moderate to good correlations with the reference measurements but had large MAE, RMSE, and NRMSE for all smoke impacted times (Figure 2, Table 5). All sensors maintained a linear response, even at concentrations as high as 200 µg/m^3^, but reported higher concentrations than the reference with average slopes of 1.27 for the RAMP, 1.35 for the AQY, and 2.03 for the PA, CF = 1 (1.37, CF = atm) during smoke impacted times. The slopes for each sensor differed from the reference (*p* < 0.001) and differed from each other (*p* < 0.001). The high bias was not the same for each fire, although some of this variation may be due to the different reference instruments operated at each measurement location and the different concentration ranges, as discussed below. The highest MBE was observed at the Natchez fire where the concentrations were greatest. At the lowest concentration range (1–10 µg/m^3^), the sensors exhibited a low bias (0.5–0.9) compared to the reference. 

The PA PM_2.5_ (both CF = atm and CF = 1) measurement showed the highest correlation with the reference of all the sensors evaluated here (Table 5), with r^2^ greater than 0.95 for all sites, except for the Pinehurst location at the Alder fire. At the Pinehurst site, all sensors exhibited a low r^2^, that was statistically significant (*p* < 0.001), but suggested other factors were contributing to a variable sensor response The AQY PM_2.5_ measurement had the poorest correlation with the reference measurement, with r^2^ values ranging from 0.52 (Pinehurst site, Alder Fire) to 0.77 (Natchez fire), but these correlations were still statistically significant (*p* < 0.001). 

Like all the sensors, the PA had the highest correlation to the reference PM_2.5_ measurement when the concentrations were very high. The PA deviated from the linear relationship with the reference at the highest concentrations indicating that the sensor may begin saturating at PM_2.5_ concentrations greater than 200 μg/m^3^. The two PA channels a and b were nearly identical (e.g., slope = 1.03, *p* < 0.001, r^2^ = 0.999, *p* < 0.001 at the Natchez fire). 

Despite using the same underlying PM_2.5_ sensor (PMS5003) as the PA, the RAMP concentrations were lower than the PA and exhibited a lower slope and lower r^2^ (Figure 2, Table 5). These differences may be due to the sensor package design, e.g., different PM_2.5_ inlets or thermal characteristics, or due to different post-processing of the sensor output by the RAMP compared to the essentially raw sensor output from the PA. However, there was limited data from collocated RAMP and PA sensors during smoke impacted times available for comparison. The two models operated side by side for almost six days at the Alder fire–Pinehurst site (maximum PM_2.5_ = 32 µg/m^3^) and five hours at the AIRS site when impacted by prescribed fire smoke (maximum PM_2.5_ = 40 µg/m^3^). The RAMP reported statistically significant lower concentrations than the PA at the Pinehurst site (34% lower) but was not statistically different at the AIRS prescribed fire (11% lower). The PA and RAMP were significantly correlated (*p* < 0.001) with one another during smoke impacted measurements (r^2^ of 0.99 and 0.81 at the two sites), and similarly correlated well at lower ambient concentrations measured during long term monitoring at AIRS (r^2^ = 0.92, *p* < 0.001 and normalized MBE of −2%).

### 3.4. Factors Impacting Sensor Performance

#### 3.4.1. Sensor Performance—Accuracy, Precision, Linearity

All sensors exhibited a high bias, consistent with other field evaluations of the PA [7,16,21,29,30] and other optical-based sensors [8,15,16,19]. Additionally, the sensors evaluated here had higher correlations to the reference than most field studies using optical-based sensors (0.3 < r^2^ < 0.95) [10]. This was likely due to the much higher PM concentrations encountered near fires, where many more hourly averages are above the lower detection limits of both the sensors and the reference instruments [21]. Sensor performance during smoke impacted times was more similar to the high correlations observed in laboratory evaluations, where the concentration range is much larger and aerosol characteristics are roughly constant [14,16,19]. The sensors evaluated here also exhibited a linear response up to the very unhealthy AQI level (150.5–250.5 µg/m^3^), meaning that with a simple linear correction these sensors may be useful for identifying appropriate health guidance during wildfires. Our results are consistent with laboratory sensor evaluations that show a linear response up to 100–500 µg/m^3^, depending on the sensor [12,14]. Although we did not observe higher concentrations, laboratory evaluations of the PMS5003 (sensor in the PA and RAMP) show that a polynomial calibration may be more appropriate above PM_2.5_ concentrations of ~500 µg/m^3^ and that the sensor has an upper measurement limit of 10,000–10,000 µg/m^3^ [12,14]. This suggests that the PA and RAMP with a polynomial correction may provide accurate PM_2.5_ measurements into the hazardous AQI (>250.5 µg/m^3^) and above any public health guidance action level.

The impact of sensor age on performance was estimated by comparing the ratio of PM_2.5_ concentration measured by the sensor to the reference vs. the cumulative PM_2.5_ concentration in µg/m^3^. This was done for the RAMP at the Alder fire, which repeatedly experienced high smoke concentrations and the PA at the AIRS site, which had the longest operating time. The PA ratio slightly decreased with PM_2.5_ exposure (slope = −5.6 × 10^−6^, r^2^ = 0.03, *p* < 0.001) and the RAMP ratio slightly increased (slope = 1.33 × 10^−5^, r^2^ = 0.06, *p* < 0.001). The impact of sensor age was found to be minimal, but further study is needed over multiple fire seasons to determine the impact of high concentrations of smoke on sensor performance.

#### 3.4.2. Smoke Specific Correction

The linear regression parameters varied when calculated individually for each field deployment, but the limited sample size at the highest concentrations can have a disproportionate effect on the regression. Overlapping the sensor and reference comparison data from each location shows a similar relationship between the sensor concentration and the reference and suggests a single correction might be widely applied during smoke impacted times (Figure S3). We combined all the smoke impacted datasets for each sensor and performed a linear regression to derive a smoke calibration. Applying this correction to each dataset reduced the MAE and the NRMSE for all sensors. The largest error reductions were for the AQY; the correction reduced the MAE by 77% and the NRMSE by 81% (Table 6). Including a correction for environmental conditions further improved the comparison between the AQY and the reference, with the NRMSE decreasing from 31 to 25%. The PA and RAMP also saw large reductions in error with the correction, but the impact of including temperature, RH, or both caused a minimal further reduction of the error. The RAMP has a correction factor (β) about 12% higher than the PA, demonstrating that sensor package-specific correction is still useful even if the using same OEM sensor.

**Figure 3:**
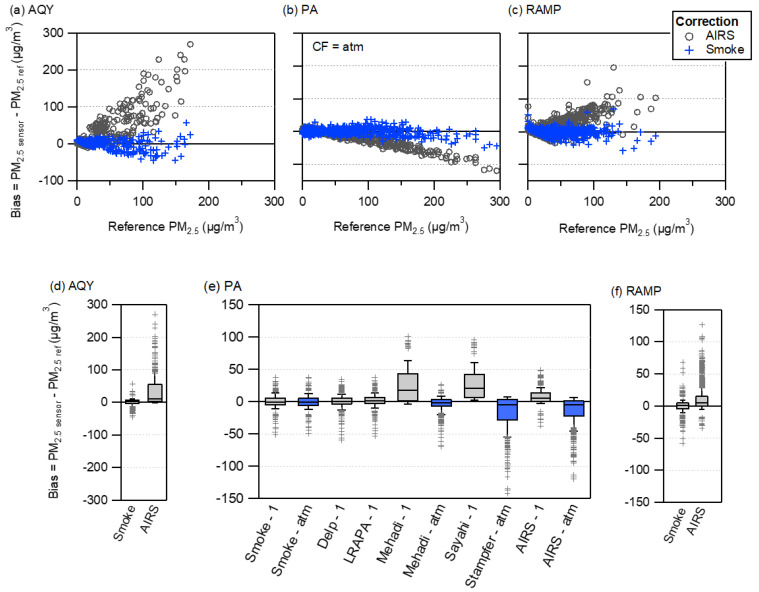
Bias for the AIRS ambient and smoke corrected sensor measurements for the (**a**) AQY (**b**) PA and (**c**) RAMP. Box plots of the hourly bias values for different correction equations for the (**d**) AQY (**e**) PA and (**f**) RAMP. ‘Smoke’ correction is from all smoke impacted sites, ‘AIRS’ correction is for ambient in NC, PA corrections from the literature are from smoke impacted Delp and Singer [23], Lane Regional Air Programs Association (LRAPA) [31], Mehadi et al. [7], Sayahi et al. [8], and Stampfer et al. [32]. ‘-1’ and ‘-atm’ refer to the data source the correction was applied to and was based off current understanding of the data source used to generate each correction.

The optimal smoke correction for each sensor was determined as simple linear correction without environmental parameters (C and **β** only in Table 2) because it reduced NRMSE and did not require other measurements (temperature and RH) which periodically failed for some of the sensors. The hourly bias versus reference PM_2.5_ concentration for the smoke correction compared to the correction developed from ambient measurements at AIRS is shown in Figure 3a–c. Using a correction developed from ambient conditions (mean PM_2.5_ concentration of 7.7 µg/m^3^) resulted in a high bias for all sensors. The AQY was most strongly impacted with errors as large as 300 µg/m^3^ at the highest smoke concentrations (Figure 3a,d). The bias was much lower for the PA with ambient correction only increasing the median bias by 6.9 µg/m^3^ for CF = 1 and decreasing by 4.2 µg/m^3^ for CF = atm. Although the smoke correction was more accurate, an ambient correction may provide reasonable results for smoke impacted PA concentrations. The bias for smoke corrected PA data shows a clear nonlinear response (Figure 3b). There is a decreasing trend in the bias at 200 µg/m^3^, which does not strongly impact bias until above 300 µg/m^3^. There is insufficient data above 200 µg/m^3^ to develop a polynomial correction, which has been identified as the best fit for high concentration laboratory measurements [11,14].

We compared our smoke correction factor with published corrections for PA or PMS5003 for wildfire smoke [8,23] or woodsmoke [7,31,32]. (Figure 3e). There are no published corrections for the AQY or RAMP under smoke impacted conditions for comparison. There is good agreement among some corrections as evidenced by near-zero bias, similar to our smoke correction. The correction by Delp and Singer [23] is nearly identical to our smoke correction, with a median bias of 0.58 µg/m^3^. Their correction factor was similarly derived from measurements at sites across CA impacted by several wildfires in 2018. Alternatively, the Sayahi el al. [8] PMS5003 correction developed from wildfire smoke impacted measurements in central Utah exhibits a high bias of 21 µg/m^3^. Mehadi et al. [7] also developed a correction from laboratory simulated wildfire smoke, but concentrations were all above 250 µg/m^3^ in the range where the PA response is nonlinear. Mehadi et al. [7] noted the nonlinear PA response and a correction derived from their linear fit of the data had an intercept of 928 µg/m^3^, which resulted in very large errors and so was not included in Figure 3.

The Stampfer et al. [32] (using a PMS5003), Mehadi et al. [7], and LRAPA corrections were all developed in woodsmoke impacted areas during the wintertime. Again, some corrections perform well (e.g., LRAPA at 1.0 µg/m^3^), while Mehadi et al. [7] have a high bias (17.3 µg/m^3^) and Stampfer et al. [32] exhibit a low bias (−4.9 µg/m^3^). The mixed agreement across studies could be due to the different sensors used (PMS5003 vs. PA), but also due to the confusion surrounding the Plantower correction factor (CF = 1 vs. CF = atm). For example, Mehadi et al. [7] did not report which CF was used in their correction; when applying their correction to CF = 1 data the median bias is 17.3 and changes to −2.8 when applied to CF = atm data. Even when the CF is specified as in Sayahi et al. [8] and Stampfer et al. [32] the median bias is 21.0 and −4.9 respectively. These different results may be due to differences between the PA used here and the PMS5003 used in these studies or from using the wrong CF data (CF labels were swapped in the PA compared to the PMS5003 in firmware versions released before fall of 2019). The LRAPA correction was originally listed on the PurpleAir Map as being applicable to CF = 1, but in August 2020 was changed to CF = ATM. A complete reanalysis of all PA data may be warranted to ensure corrections developed from different datasets are using the same CF.

Another factor explaining the differences between corrections could be the different smoke properties from laboratory simulations and woodsmoke. Mehadi et al. [7] showed the PA was sensitive to the fraction of elemental carbon in the PM and had a lower PA/reference ratio (translates to higher β) when elemental carbon content was higher. Laboratory simulations have shown that biomass burning emissions can have varying ratios of elemental carbon to PM, depending on the fuel type and combustion conditions [33]. Although elemental carbon is generally a minor fraction of the PM from fires, even small changes in PM elemental carbon content can impact PM optical properties [34]. However, the agreement in our study and that by Delp and Singer [23] across different fires and locations suggests that a constant correction equation for wildfire smoke would provide sensor derived PM_2.5_ concentrations with minimal error (<20% and 10 µg/m^3^). A uniform smoke calibration for optical sensors may be reasonable given the mostly invariant particle properties of aged smoke. PM from most wildfires is almost entirely composed of organic carbon (>90% by mass), although certain combustion conditions (e.g., flaming) and fuel types (e.g., grasses) can have larger fractions of black carbon (or elemental carbon) and inorganic species that would impact optical properties [3]. Freshly emitted smoke has a lognormal particle size distribution with a median diameter in the range of 100–150 nm and geometric standard deviation of 1.6−1.9 [35], which is below the limit of detection of most optical sensors. After several hours in the atmosphere, the particle size increases and the distribution narrows to a median diameter range of 175–300 nm and geometric standard deviations of 1.3–1.7 [35,36], typical of aged smoke [37]. Although, optical sensors detect only a portion of the particle size distribution, the near-uniform median size and particle composition results in a constant correction relationship to account for what optical sensors do not detect. The smoke correction may also provide reasonable results for other ambient environments typical to many areas in the U. S. that are also dominated by organic carbon PM [38,39,40], with sizes ranging from 100–500 nm [41].

#### 3.4.3. Impact of Meteorological Conditions

Optical sensors have well-documented overestimation of PM_2.5_ concentrations at elevated RH [20]. At the AIRS site, where a broad RH range was experienced (average temperature 20 ± 8 °C and RH about 80 ± 17%), the correlation with the reference for the AQY increased when RH was included in the linear regression, but not for the PA and the RAMP (Table S3). This may be due to the sensor design (e.g., light source or scattering angle) or package configuration (e.g., thermal characteristics, etc.). For example, the PA has been shown to be relatively insensitive to RH, which may be due to the internal RH consistently reporting considerably lower than ambient RH [7]. Including terms for temperature and RH in the smoke correction also did not improve the r^2^ or the MAE or NRMSE for the PA or RAMP but did cause slight improvements for the AQY (Table 6). RH was lower near fires and generally under 60% during smoke impacted times, although the RH ranged from 10–100% over all fires (Table S2). The sensor insensitivity to RH may be due to the lower RH values near the fire. The RH during our smoke sampling was generally below 80%, whereas a significant number of samples >75% would be needed to address the range of RH most relevant for when light scattering methods are most susceptible to errors due to humidity. The particle properties also play a role. Zamora et al. [19] attributed the Plantower PM3003 insensitivity to RH when exposed to incense smoke to the low hygroscopicity of these particles; this may be true for wildfire smoke which also has low hygroscopicity [42]. For the sensors tested here, a simple linear regression without environmental terms was sufficient to reduce MBE to less than 10 µg/m^3^.

#### 3.4.4. Impact of Reference Measurement

Some of the variations in the sensor comparison with the reference may be due to the reference measurement itself. Only the AIRS, Springville, and Pinehurst sites had an FEM of varying measurement techniques (beta-attenuation gauge—BAM—or light scatter—EDM180). The other monitors used as references (EBAM, E-Sampler), as described by the manufacturer, were consistent with the FEM designation requirements but had not been designated as an FEM and are commonly used to measure air quality impacts from wildfire smoke. 

PM_2.5_ measurement during smoke impacted times represents a unique measurement challenge that is not explicitly addressed in the federal reference and equivalency method designations. For example, the high organic PM loadings that occur during smoke can evaporate from the federal reference method (FRM) samples and lead to a low bias [43]. Although FEMs are required to be validated against FRM filter samples at concentrations ranging from 3 to 200 µg/m^3^ at multiple locations across the U.S. [26], this does not specifically include smoke impacted times, where the concentrations can be much greater. The performance of FEMs and near-FEM grade instruments during these high pollution times have not been validated in the field. For example, Schweizer et al. [44] found that the EBAMs commonly used for temporary smoke monitoring networks overreported PM_2.5_ compared to BAMs, but only when RH was above 40%. These potential variations in the reference measurement accuracy and precision during smoke impacted times may have led to weaker correlations and introduced variation in the slope of the linear regressions across sites. The magnitude of these effects is difficult to quantify.

## 4. Conclusions

Low-cost PM sensors provide accurate smoke PM_2.5_ concentrations with appropriate correction. A linear correction was effective at reducing error to less than 25% for the AQY, 16% for the PA, and 27% for the RAMP and did not vary across the fires studied here. Corrections developed under ambient conditions did not reduce the error as much as one developed from smoke impacted conditions, resulting in bias ranging from −150 to 300 µg/m^3^. Using a smoke-specific correction, low-cost sensors can report PM_2.5_ concentrations that are comparable to current smoke monitoring networks and because of their lower cost, they may be deployed in large numbers. They hold the potential to greatly increase our knowledge of the temporal and spatial variation of smoke and this information can be used to provide public health guidance.

More studies on sensor performance for monitoring wildfire smoke are still needed, particularly as sensor technology advances, since each package may have a differing performance to smoke. Evaluations are also needed at higher concentrations as optical-based sensors can saturate when the concentrations are very high. The sensors assessed here had linear correlations with reference monitors up to approximately 200 µg/m^3^, but PM_2.5_ concentrations near fires can greatly exceed this level and accurate measurement at these high concentrations are needed to determine if personal protective equipment—especially for outdoor workers—or even evacuations for smoke hazards should be considered.

## Figures and Tables

**Table 1:** List of deployment sites and collocated particulate matter (PM) measurements.

Event	Location	Dates	Sensors	Reference Instrument	PM Source
AIRS	RTP, NC	8/8/2018–6/30/2019	AQY, PA, RAMP	EDM 180 (GRIMM)	Ambient, Prescribed fire
Natchez Fire	Happy Camp, CA	8/11–8/29/2018	AQY, PA	E-BAM (Met One)	Wildfire
Bald Mt./Pole Creek Fire	Price, UT Dutch John, UT	9/24–10/1/2018	AQY, PA	E-SAMPLER (Met One)	Ambient
Alder Fire	Springville, CA	10/19–11/27/2018	RAMP	BAM 1020 (Met One)	Wildfire
	Pinehurst, CA	10/20–10/27/2018	AQY, PA, RAMP	BAM 1020 (Met One)	Prescribed fire/ Wildfire
	Camp Nelson, CA	10/20–10/27/2018	RAMP	E-BAM (Met One)	Wildfire

**Table 2:** Average temperature linear regression parameters ^a^ for 2 sensors of each type at AIRS during ambient monitoring.

Sensor	Temperature Collocation Dates	N (hr)	Slope	Intercept	R^2^	MBE (°C)	NRMSE (%)	PD_avg_ (%)
AQY	8/9/18–12/3/18	1197	1.19	−2.29	0.98 *	1.83	12	1.1
PA	8/10/18–4/30/19	5454	0.9	7.2	0.91 *	5.23	34	6.1
RAMP	12/12/18–7/31/19	4893	1.14	−0.83	0.96 *	1.36	19	4.8

^a^ PM_2.5 sensor_ = Intercept + Slope PM_2.5 reference,_ * Statistically significant at *p* < 0.01.

**Table 3:** Average relative humidity linear regression parameters ^a^ for 2 sensors of each type at AIRS during ambient monitoring.

Sensor	Relative Humidity Collocation Dates	N (hr)	Slope	Intercept	R^2^	MBE (%)	NRMSE (%)	PD_avg_ (%)
AQY	8/9/18–12/3/18	1197	1.12	−16.9	0.95 *	−4.90	11	11.4
PA	8/10/18–4/30/19	4654	0.57	5.29	0.84 *	−24.30	37	4.0
RAMP	12/12/18–7/31/19	4893	0.90	2.23	0.95 *	−4.10	10	2.2

^a^ PM_2.5 sensor_ = Intercept + Slope PM_2.5 reference,_ * Statistically significant at *p* < 0.01.

**Table 4:** Average PM_2.5_ linear regression parameters ^a^ for 2 sensors of each type at AIRS during ambient monitoring.

Sensor	PM_2.5_ Collocation Dates	N (hr)	Slope	Intercept	R^2^	MBE (µg/m^3^)	NRMSE (%)	PD_avg_ (%)
AQY	8/9/18–10/18/19	1186	0.89	−0.21	0.37 *	−0.01	58	13.4
PA CF = atm	8/10/18–4/30/19	4654	1.61	−1.40	0.86 *	2.89	66	6.9
PA CF = 1	8/10/18–4/30/19	4654	1.63	−1.51	0.86 *	2.92	67	6.9
RAMP	12/12/18–7/31/19	3041	0.88	2.45	0.92 *	1.64	34	6.7

^a^ PM_2.5 sensor_ = Intercept + Slope PM_2.5 reference,_ * Statistically significant at *p* < 0.01.

**Table 5:** Comparison statistics and linear regression parameters ^a^ for each data set.

Sensor	Location	N (hr)	Slope	Intercept	r^2^	MBE (µg/m^3^)	NRMSE (%)
AQY	AIRS—Ambient	2815	0.84	−0.14	0.45 *	−1.39	53
	AIRS—Prescribed Fire	-	-	-	-	-	-
	Natchez	181	2.18	−7.89	0.86 *	63.89	146
	Pole Creek	63	0.54	0.87	0.77 *	−1.18	45
	Alder Pinehurst	136	1.35	1.40	0.52 *	5.91	82
PA (CF = atm)	AIRS—Ambient	4750	1.61	−1.46	0.87 *	2.98	66
	AIRS—Prescribed Fire	10	1.61	−2.49	1.00 *	6.16	70
	Natchez	367	1.20	15.23	0.96 *	32.82	44
	Pole Creek	88	0.93	0.36	0.74 *	0.13	50
	Alder Pinehurst	161	1.30	9.78	0.62 *	13.79	117
PA (CF = 1)	AIRS—Ambient	4750	1.63	−1.58	0.87 *	3.00	48
	AIRS—Prescribed Fire	10	2.44	−7.97	0.99 *	12.36	154
	Natchez	367	1.85	18.41	0.96 *	91.17	125
	Pole Creek	88	0.93	0.36	0.74 *	0.13	50
	Alder Pinehurst	161	1.81	4.76	0.62 *	15.67	145
RAMP	AIRS—Ambient	3493	0.89	2.58	0.91 *	1.83	37
	AIRS—Prescribed Fire	10	1.35	−0.05	0.99 *	4.89	15
	Natchez	-	-	-	-	-	-
	Pole Creek	-	-	-	-	-	-
	Alder Pinehurst	107	0.77	6.61	0.69 *	3.69	5
	Alder Springville	802	1.48	−2.48	0.85 *	14.47	3

^a^ PM_2.5 sensor_ = Intercept + Slope PM_2.5 reference_ * Statistically significant at *p* < 0.01.

**Table 6:** Smoke correction linear regression parameters ^a^, adjusted r^2^, MAE, and NRMSE. Optimal smoke correction in bold.

Sensor	C	β	β_T_	β_RH_	Adjusted r^2^	MAE (µg/m^3^)	NRMSE (%)
AQY					0.90	39.4	167.1
	**7.56**	**0.41**			**0.90**	**8.90**	**31.0**
	13.48	0.42	−0.327		0.91	8.89	30.3
	8.71	0.41		0.429	0.93	8.13	26.9
	−36.7	0.38	0.809	0.782	0.93	7.52	25.1
PA (cf = atm)					0.97	26.3	52.2
	−**7.96**	**0.79**			**0.97**	**7.68**	**18.7**
	−16.06	0.79	0.351		0.97	7.30	16.0
	−1.93	0.80		−0.206	0.97	7.37	16.2
	−13.68	0.79	0.300	−0.041	0.97	7.29	16.0
PA (cf = 1)					0.97	66.2	143.3
	−**3.21**	**0.51**			**0.97**	**7.61**	**16.9**
	−9.43	0.51	0.270		0.97	7.49	16.6
	3.18	0.52		−0.216	0.97	7.36	16.4
	3.27	0.52	−0.002	−0.218	0.97	7.36	16.4
RAMP					0.89	15.8	80.5
	−**1.38**	**0.57**			**0.89**	**6.40**	**28.3**
	−0.94	0.57	0.164		0.90	6.27	28.0
	−3.16	0.56		−0.063	0.90	6.28	27.7
	−2.54	0.57	0.135	−0.028	0.90	6.22	27.6

^a^ PM_2.5_ = C + β sensor PM_2.5_ + β_T_ sensor Temperature (°) + β_RH_ sensor RH (%).

## Supplementary Materials

The following are available online at https://www.mdpi.com/1424-8220/20/17/4796/s1, Figure S1: Typical sensor package setup, S2: (a) Scatter plot of CF = atm to CF = 1 PM_2.5_ concentrations and (b) ratio of the CF = atm to CF = 1 vs. PM_2.5_ concentrations vs. PM_2.5_ CF = 1 concentration at the Natchez fire, S3: Overlaid scatter plot of smoke impacted datasets with the linear fit smoke calibration and the resulting MAE and NRMSE for the calibration adjusted data for (a) AQY (b) PA and (c) RAMP. Table S1: Selected sensor manufacturers, models, and measured parameters, Table S2: Reference mean (µ), standard deviation (σ), minimum value (min), and maximum value (max) for fine particulate matter (PM_2.5_) concentrations and temperature (T) and relative humidity (RH) at each deployment location, Table S3: Linear regression parameters for sensor correction and adjusted R^2^ developed from AIRS ambient evaluation.

## Appendix A. Data Analysis Formulas

The percent difference was calculated when only two sensors of the same type were collocated for each hour and averaged over the entire dataset (PD_avg_):

(A1) $$PD_{avg}=\;\frac{1}{N}\overset{N}{\underset{i}{\sum }}\frac{{\left|PM_{2.5-1}-PM_{2.5-2}\right|}_{i}}{0.5\;{\left(PM_{2.5-1}+PM_{2.5-2}\right)}_{i}}\times 100\%$$

where PM_2.5-1_ and PM_2.5-2_ is the hourly PM_2.5_ concentration measured by Sensors 1 and 2, respectively and N is the number of hours in the entire dataset.

The effect of the correction factors was determined by calculating the mean bias error (MBE):

(A2) $$MBE=\;\frac{1}{N}\overset{N}{\underset{i}{\sum }}{\left(X_{sensor}-\;X_{reference}\right)}_{i}$$

the mean absolute error (MAE):

(A3) $$MAE=\frac{1}{N}\overset{N}{\underset{i}{\sum }}\;{\left|X_{sensor}-\;X_{reference}\right|}_{i}$$

and the root mean square error (RMSE):

(A4) $$RMSE=\sqrt{\frac{1}{N}\overset{N}{\underset{i}{\sum }}{\left(X_{sensor}-\;X_{reference}\right)}_{i}^{2}}$$

where X_sensor_ is the quantity (e.g., temperature or PM_2.5_ concentration) averaged over each hour measured by the sensor, X_reference_ is the same quantity measured simultaneously by the reference instrument, and N is the total number of matching hours (i) of sensor and reference. Hours with missing sensor data were removed from the analysis. The normalized root mean square error (NRMSE) was also calculated by dividing the RMSE by the mean reference quantity over the entire dataset to allow for comparison with datasets of different concentration ranges.
