# Supplementary material for: Field Evaluation of Low-Cost Particulate Matter Sensors for Measuring Wildfire Smoke
Source: Sensors (Basel). 2020 Aug 25;20(17):4796. doi: 10.3390/s20174796 (PMC7506753; doi:10.3390/s20174796)
Supplement: Supplementary file 1 [file sensors-20-04796-s001.pdf]

Supplemental Information

Field evaluation of Low-Cost Particulate Matter Sensors for Measuring Wildfire Smoke

Amara L. Holder[1]*, Anna K. Mebust[2], Lauren A. Maghran[2], Michael R. McGown[3], Kathleen E. Stewart[2], Dena M. Vallano[2], Robert A. Elleman[3], Kirk R. Baker[4]

1 US Environmental Protection Agency, Office of Research and Development; holder.amara@epa.gov
2 US Environmental Protection Agency, Region 9; mebust.anna@epa.gov, maghranlauren@gmail.com, stewart.kathleen@epa.gov, vallano.dena@epa.gov
3 US Environmental Protection Agency, Region 10; mcgown.michael@epa.gov, elleman.robert@epa.gov
4 US Environmental Protection Agency, Office of Air Quality Planning and Standards; baker.kirk@epa.gov
* Correspondence: holder.amara@epa.gov; Tel.: +1-915-541-4635

Received: 02 August 2020; Accepted: 21 August 2020; Published: date

Number of Pages: 7
Number of Figures: 3
Number of Tables: 3

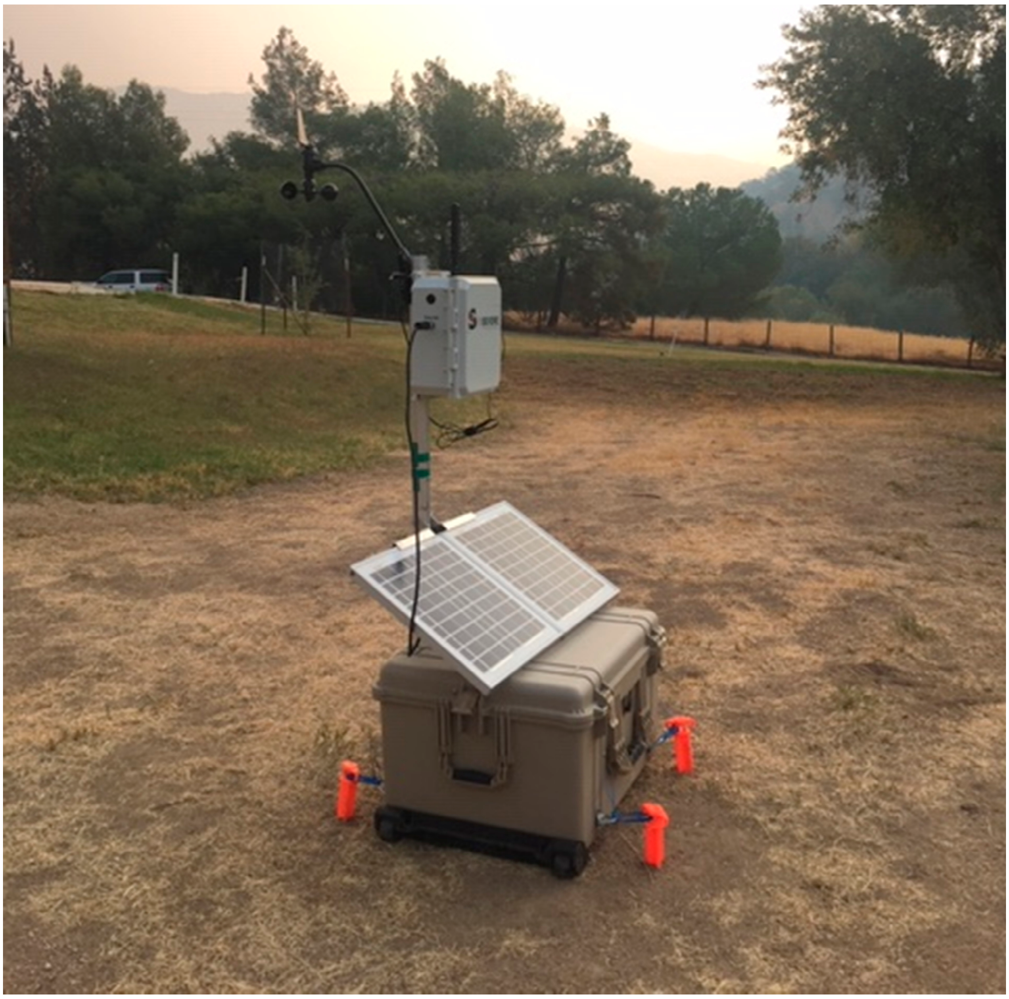

Figure 1. Typical sensor package setup.

(a)

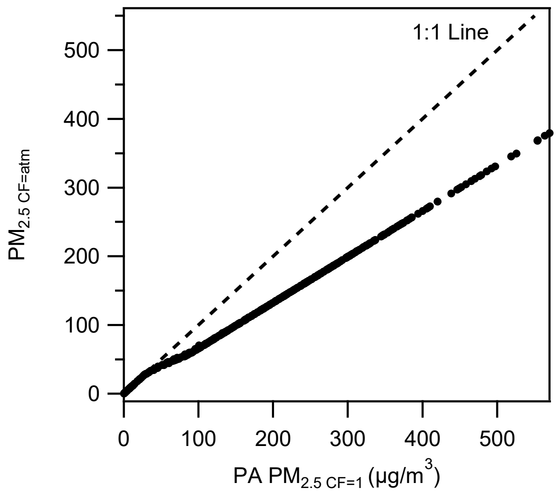

(b)

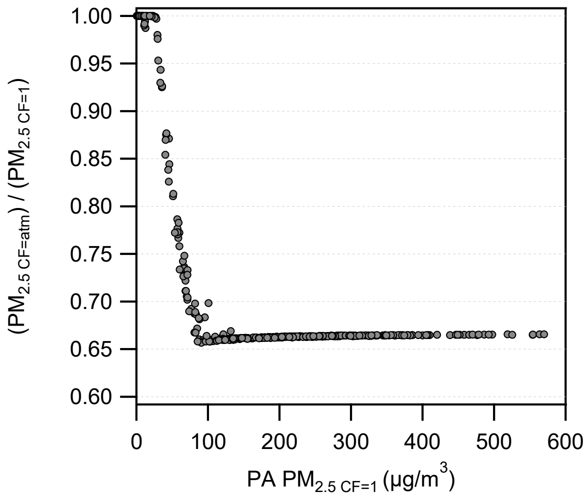

Figure S2. (a) Scatter plot of CF=atm to CF = 1 $PM_{2.5}$ concentrations and (b) ratio of the CF = atm to CF = 1 vs $PM_{2.5}$ concentrations vs $PM_{2.5}$ CF = 1 concentration at the Natchez fire.

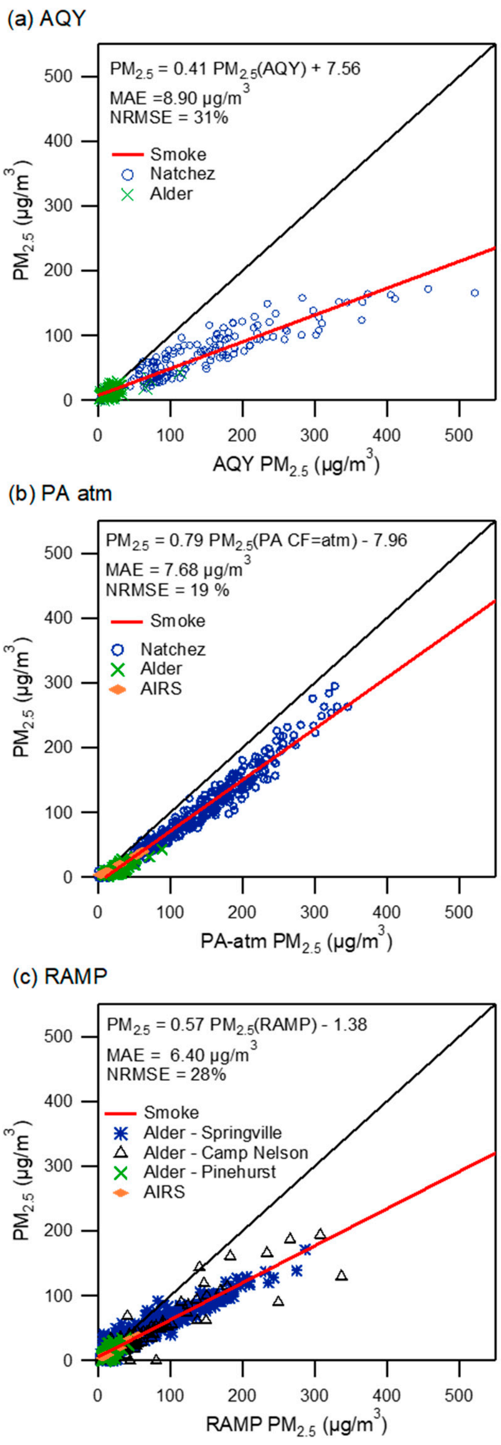

Figure S3. Overlaid scatter plot of smoke impacted datasets with the linear fit smoke calibration and the resulting MAE and NRMSE for the calibration adjusted data for (a) AQY (b) PA and (c) RAMP.

Table 1. Selected sensor manufacturers, models, and measured parameters.

Sensor Manufacturer	Model	Pollutant Capability	PM Sensor Model	Additional Measurements
SenSevere	Real Time Affordable Multi-Pollutant Monitor (RAMP)	$PM_{2.5}$, CO, CO_2	Plantower PMS5003	Relative Humidity<br>Temperature<br>Wind speed/direction
Aeroqual	Micro air quality station (AQY)	$PM_{2.5}$, NO_2, O_3	Novafitness SDS011	Relative Humidity<br>Temperature
PurpleAir	PA-IISD (PA)	PM_1, $PM_{2.5}$, PM_{10}	Plantower PMS5003	Relative Humidity<br>Temperature<br>Pressure

Table S2. Reference mean (μ), standard deviation (σ), minimum value (min), and maximum value (max) for fine particulate matter ($PM_{2.5}$) concentrations and temperature (T) and relative humidity (RH) at each deployment location

Deployment	$PM_{2.5}$ (μg/m³)				T (°C)				RH (%)			
	μ	σ	Min	Max	μ	σ	Min	Max	μ	σ	Min	Max
AIRS ambient	7.7	4.3	0.5	102.6	19.1	8.4	-4.5	33.7	80.0	17.6	23.4	100
AIRS Prescribed Fire	16.4	16.7	3.6	51.6	26.28	1.4	20.7	26.3	41.6	5.2	34	53
Natchez Fire	86.4	62.8	0	295								
Bald Mt./ Pole Creek Fire	3.4	3.0	0	9	19.0	7.2	5.6	32.1	20.3	9.3	5	48
Alder Fire/ Springville	35.2	28.4	-5	291	11.7	5.1	2.7	22.6	46.9	22.3	12.3	100.2
Alder Fire/ Pinehurst	13.0	6.6	2	43	15.5	4.3	10	24.4	40.1	8.8	18.5	62

Table S3. Linear regression parameters[a] for sensor correction and adjusted R^2 developed from AIRS ambient evaluation.

Sensor	C	β	$β_T$	$β_{RH}$	Adjusted R^2
AQY	4.45	0.65			0.48
	6.81	0.71	-0.127		0.54
	-2.35	0.67		0.083	0.64
	-1.71	0.70	-0.038	0.081	0.64
PA (CF = atm Lower)	1.74	0.53			0.86
	2.08	0.54	-0.030		0.87
	0.44	0.52		0.020	0.87
	0.80	0.52	-0.027	0.020	0.88
PA (CF=1 Higher)	1.79	0.53			0.86
	2.26	0.54	-0.042		0.86
	0.43	0.51		0.021	0.87
	0.93	0.52	-0.038	0.020	0.88
RAMP	-1.38	0.97			0.85
	-0.94	1.03	-0.076		0.88
	-3.16	0.94		0.032	0.88
	-2.54	0.98	-0.054	0.026	0.90

[a] $PM_{2.5} = C + β$ sensor $PM_{2.5} + β_T$ sensor T + $β_{RH}$ sensor RH
